# Policy Implications of Genetic Information on Regulation under the Clean Air Act: The Case of Particulate Matter and Asthmatics

**DOI:** 10.1289/ehp.8299

**Published:** 2005-10-26

**Authors:** C. Bradley Kramer, Alison C. Cullen, Elaine M. Faustman

**Affiliations:** 1 Center for the Study and Improvement of Regulation; 2 Daniel J. Evans School of Public Affairs, and; 3 Institute for Risk Analysis and Risk Communication, Department of Environmental and Occupational Health Sciences, School of Public Health, University of Washington, Seattle, Washington, USA

**Keywords:** asthma, Clean Air Act, genetics, particulate matter, risk analysis

## Abstract

The U.S. Clean Air Act (CAA) explicitly guarantees the protection of sensitive human subpopulations from adverse health effects associated with air pollution exposure. Identified subpopulations, such as asthmatics, may carry multiple genetic susceptibilities to disease onset and progression and thus qualify for special protection under the CAA. Scientific advances accelerated as a result of the ground-breaking Human Genome Project enable the quantification of genetic information that underlies such human variability in susceptibility and the cellular mechanisms of disease. In epidemiology and regulatory toxicology, genetic information can more clearly elucidate human susceptibility essential to risk assessment, such as in support of air quality regulation. In an effort to encourage the incorporation of genomic information in regulation, the U.S. Environmental Protection Agency (EPA) has issued an *Interim Policy on Genomics*. Additional research strategy and policy documents from the National Academy of Science, the U.S. EPA, and the U.S. Department of Health and Human Services further promote the expansion of asthma genetics research for human health risk assessment. Through a review of these government documents, we find opportunities for the inclusion of genetic information in the regulation of air pollutants. In addition, we identify sources of information in recent scientific research on asthma genetics relevant to regulatory standard setting. We conclude with recommendations on how to integrate these approaches for the improvement of regulatory health science and the prerequisites for inclusion of genetic information in decision making.

Since 1990, the Human Genome Project and subsequent technologic advances have made generating genetic information cheaper, easier, and more reliable, thus changing the face of science (Decaprio 1997). Recently developed technologies have enabled scientists to identify mutations that define human variability, determine the prevalence of identified genetic mutations in the population, and interpret the function and role of specific genes in disease. In May 2002, the U.S. Environmental Protection Agency’s (EPA) Science Policy Council released an *Interim Policy on Genomics* (U.S. EPA 2002c). The U.S. EPA continued to explore the application of genomic information in a second document, *Potential Implications for Genomics for Regulatory and Risk Assessment Applications at EPA* (U.S. EPA 2004b). The interim policy heralds the potential of genomic information “to enhance its assessments and better inform the decision-making process.” Genomic information has the potential to improve the U.S. EPA’s regulatory process in a key context—the setting of health-based standards directed at protecting susceptible subpopulations. The interim policy concludes that as the U.S. EPA “gains experience in applying genomics information . . . it will develop guidance to explain how genomics data can be better used in decision making, and related ethical, legal, and social implications” (U.S. EPA 2002c).

In this commentary we examine opportunities within current policy for the inclusion of genetic information in regulation of air pollutants, with particular attention to particulate matter (PM). We focus on key polymorphisms that identify asthmatics, an established sensitive subpopulation that stands to benefit from the inclusion of genetic information in air quality regulation (U.S. Senate 1970). In a subsequent analysis (Cullen AC, Kramer CB, Faustman E, unpublished data) we extend the integration of genomic science and regulatory policy using a decision analytic framework. An additional manuscript (Bradley A, Cullen A, Burke W, Faustman E, unpublished data) addresses both the importance and the challenge of incorporating genetic information in other statutory contexts, such as food safety and pesticides.

The U.S. Clean Air Act (CAA) requires that the EPA set National Ambient Air Quality Standards (NAAQS) for six criteria pollutants: carbon monoxide, lead, nitrogen dioxide, ozone, sulfur oxides, and PM. These standards are set at levels “requisite to protect public health” with “an adequate margin of safety” [U.S. Clean Air Act Amendments (CAAA) 1990 §109(b)(1)]. The CAA further requires the U.S. EPA to consider sensitive subpopulations and the increased risk they bear as a result of exposure to criteria air pollutants [U.S. Clean Air Act Amendments 1990 §108(f)(1)(C)]. Asthmatics represent a significant and increasing subpopulation in the United States (U.S. EPA 2003). Since the Centers for Disease Control and Prevention (CDC) began reporting on the occurrence of asthma in 1980, the number of asthmatics in the United States has been steadily rising. In 2002, the CDC reported that 30.8 million people were clinically diagnosed with asthma at some point over their lifetime (CDC 2005).

Asthma is a complex disease with environmental and genetic contributions to both disease susceptibility and progression. Genomic information can increase our understanding of asthma etiology as well as individual and population predisposition to developing asthma. Exposure to airborne PM exacerbates the physiologic responses leading to asthma, such as airway inflammation, and may also increase sensitization to allergens resulting in atopy, a risk factor associated with asthma (Dockery et al. 1993; Pope et al. 1995). In an effort to improve scientific understanding of the mechanisms governing the relationship between asthma and PM exposure, government agencies have developed targeted research strategies [National Research Council (NRC) 1998, 1999, 2001, 2004; U.S. Department of Health and Human Services (DHHS) 2000; U.S. EPA 2002a] and directed substantial funding to this goal. By measuring the prevalence of genetic biomarkers, scientists can quantify the health risks borne by the most susceptible subpopulations among asthmatics, as a result of exposure to specific concentrations of PM. These data can inform the air-quality standard-setting process to protect even the most sensitive individuals from adverse health effects with an adequate margin of safety.

Through a review of these government documents we find opportunities for the inclusion of genetic information in the regulation of air pollutants. In addition, we identify sources of information in recent scientific research on asthma genetics relevant to regulatory standard setting. We conclude with recommendations about integrating laboratory-based science, in the form of genetic information, into the risk management process to improve regulatory decision making.

## Materials and Methods

To analyze the potential role of genetic information in PM regulation, we considered a range of sources pertaining to the U.S. EPA’s mandate. Initially, we reviewed the statutory language of Title I of the 1990 (CAAA)—the current, primary statute for setting air quality standards (U.S. Clean Air Act Amendments 1990). Refinement of the statutory mandate was obtained through a LexisNexis (2004) search of federal court cases providing judiciary clarification of the language and its application to PM NAAQS. In addition, we examined two key documents that define the U.S. EPA’s risk assessment approach in standard setting and the potential role of genomic information in this process. The first of these, the *Air Quality Criteria for Particulate Matter* (U.S. EPA 2004a) issued by the U.S. EPA’s Office of Research and Development, is intended to “accurately reflect the latest scientific knowledge useful in indicating the kind and extent of identifiable effects on public health or welfare” (U.S. Clean Air Act Amendments 1990; U.S. EPA 2004a). The second of these documents, the Office of Air Quality Planning and Standards (OAQPS) staff paper, was prepared by the U.S. EPA’s OAQPS after extensive peer review and approval of the criteria document (U.S. EPA 2003). The OAQPS staff paper recommends a national, population-specific standard(s) based on extensive risk assessment scenarios for diverse urban centers across the United States. We consulted the 2002 *Interim Policy on Genomics* (U.S. EPA 2002c) to assess the U.S. EPA’s anticipated expansion of its efforts to incorporate genetic information in decision making and risk assessment agency-wide. We refer to the most current final version of the PM criteria document (U.S. EPA 2004a) and the recent draft PM staff paper (U.S. EPA 2003), unless otherwise noted.

In addition, to evaluate recent progress on asthma genetics research relevant to air pollution risk assessment, we reviewed the current literature through a Medline (PubMed 2004) search on asthma, genetics, and air pollution and resultant references. We then developed and applied criteria for prioritizing health science on genetic susceptibility to asthma relevant to regulation. We established these criteria on the basis of review articles detailing current trends in linkage, association, and candidate gene studies (Bracken et al. 2002; Tabor et al. 2002). Using these criteria and examination of review articles, linkage analyses, and a genetic information database, we culled six exemplary candidate genes for further review [Borish 1999; Bracken et al. 2002; Collaborative Study on the Genetics of Asthma 1997; Cookson 1999, 2002; Daniels et al. 1996; Hakonarson and Wjst 2001; Huss and Huss 2000; Online Mendelian Inheritance in Man (OMIM) 2000; Sengler et al. 2002]. We assessed multiple association studies that link asthma to single nucleotide polymorphisms within these candidate genes. Finally, we reviewed established government research strategies and identified decision points at which asthma and/or genetics are given priority.

## Results

### The CAA and genetic information.

**Figure 1:**
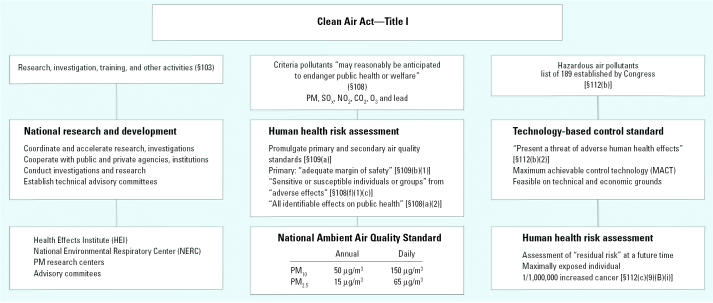
The CAA provisions for protection of human populations (Title I, U.S. Clean Air Act Amendments 1990). The CAA provides for protection of human health through research (left), standard setting for criteria pollutants (middle), and standard list of 189 HAPs, established by Congress (right).

Title I of the CAAA contains the current mandate for setting regulatory standards on air pollution (U.S. Clean Air Act Amendments 1990). In Figure 1, we highlight key language defining the statutory requirement, with a focus on PM. The left column outlines the research mandate of section 103 encouraging the development of the necessary health science data and collaboration between agencies; the middle column cites the statutory language pertaining to setting the health-based NAAQS, including PM, under sections 108 and 109; and the right column details development of technology-based standards for hazardous air pollutants (HAPs), many of which occur as PM, under section 112. In addition, the CAA requires a health-based standard for HAPs, in contexts where the technology-based standard proves insufficient to protect health. A health-based risk assessment for a HAP includes methodologies and results in conclusions also applicable to PM NAAQS (Lippmann and Schlesinger 2000). Overall, we focus on specific statutory language for criteria air pollution regulation, and the specific decision points for which the U.S. EPA’s *Interim Policy on Genomics* (U.S. EPA 2002c) recommends the incorporation of genomic information. Interpretations of “sensitive subpopulations,” “adverse effect,” and “risk assessment process,” shared by the CAA and the *Interim Policy on Genomics*, are discussed in the following sections.

#### Sensitive subpopulations.

The CAA ensures regulation that will “protect the health of sensitive or susceptible individuals or groups” [Clean Air Act Amendments 1990 §108(f)(1)(C)]. This mandate is interpreted by the courts in several cases: *Ober v. Whitman* 2001, *American Lung Association v. EPA* 1998, and *Lead Industries Association v. EPA* 1980. Each of the cases refers back to the 1970 senate report that led to the enactment of the CAA to define susceptible subpopulations (U.S. Senate 1970). The senate report states that the CAA will address “particularly sensitive citizens such as bronchial asthmatics and emphysematics,” through the development of “ambient standards necessary to protect . . . sensitive group[s] rather than a single person in such a group.” Additionally, the current PM criteria document and PM staff paper name children, the elderly, and those with preexist-ing disease, such as chronic obstructive pulmonary disease, emphysema, and asthma, as susceptible subpopulations (U.S. EPA 2003, 2004a).

Further expansion of this mandate to include genetically susceptible subpopulations is recognized under the *Interim Policy on Genomics*, which notes “the promise [of genomics information] to identify variability and susceptibilities in individuals from exposed populations” (U.S. EPA 2002c). Provided that genetic factors regulate multiple aspects of asthma progression, researchers might ultimately differentiate between genetically predisposed asthmatic individuals to identify those most susceptible to PM exposure.

#### Adverse effects.

The CAA also seeks to identify and protect citizens from “adverse effects” caused by air pollution exposure. The PM staff paper’s human health risk assessment establishes a working definition for adverse effects, with three types identified for consideration in the NAAQS process: *a*) mortality—nonaccidental total due to both cardiovascular and respiratory causes; *b*) morbidity—hospital admissions for cardiovascular and respiratory causes; and *c*) symptomatic—increased respiratory symptoms (U.S. EPA 2003). These are adverse effects measured most consistently in epidemiologic PM exposure studies. Beyond this definition, the statutory language leaves room for interpretation that invites inclusion of any and all adverse biologic effects, including those identified with genetic information (American Thoracic Society 2000; Marchant 2002).

The *Interim Policy on Genomics* notes that “genomics methodologies are expected to provide valuable insights for considering how environmental stressors affect . . . how changes in gene expression may relate to adverse effects” (U.S. EPA 2002c). Genetic biomarkers can include biomarkers of susceptibility, as shown in Table 1, as well as indicate subclinical precursors to adverse effects as provided in genomic RNA microarray studies showing changes in gene expression profiles. The U.S. EPA has historically considered subclinical events as legitimate indicators of health effects; for example, elevations in molecular biomarkers for exposure were used as evidence of impaired biologic function in setting the 1978 NAAQS for lead (Marchant 2002). Genomic biomarkers promise to provide a substantial increase in quantifiable data that directly define adverse effects.

#### Risk assessment process.

The PM staff paper details the risk assessment process used in setting NAAQS. The current draft PM staff paper focuses on epidemiologic studies, relying solely on relative risk metrics from daily time-series population studies, while excluding personal exposure and risk data (U.S. EPA 2003). Studies based on daily measures present some challenges in the estimation of long-term exposure and risk.

To address this gap in applicable available data, the U.S. EPA performs sensitivity analyses on key assumptions in the risk assessment. For example, sensitivity analyses targeting variation in concentration response, including lag time in presentation of exposure-related health effects, long-term exposure effects, and hypothetical thresholds for PM concentration response, are performed. The current PM staff paper states: “There are, of course, several other significant uncertainties in the risk assessment. . . . If there were sufficient information to characterize these sources of uncertainty quantitatively, they could be included in a Monte Carlo analysis to produce confidence intervals that more accurately reflect all sources of uncertainty” (U.S. EPA 2003).

Genetic information could be used to improve sensitivity analyses. In fact, the U.S. EPA tacitly approves the immediate use of genetic information in risk-based regulation; however, a number of barriers are evident. The *Interim Policy on Genomics* states that “while genomics data may be considered in decision making at this time, these data alone are insufficient as a basis for decisions” and will be considered only on “a case-by-case basis” (U.S. EPA 2002c). Consequently, the U.S. EPA remains limited by a lack of reliable genomic data for informing decisions and effectively constructing sensitivity analyses. Successful inclusion would require findings based on exposure response in geographically or nationally representative epidemiologic models, with reproducible data on genetic responses.

### Regulatory health science relevant to asthma.

Asthma afflicts a significant and increasing fraction of the U.S. population and is the primary chronic disease in children. The CDC reports that 21.9 million (10.6%) adults suffer from clinically diagnosed asthma at some point in their lifetime (CDC 2005). Children are disproportionately likely to suffer asthma, with 8.9 million (12.2%) experiencing the onset of asthma before the age of 18. In 2002, asthmatics accounted for 13.9 million hospital outpatient visits, 1.9 million emergency department visits, 484,000 hospitalizations, and 4,261 deaths (CDC 2005). The direct and indirect costs of the disease are substantial, amounting to $12.7 billion in 1998 (Weiss and Sullivan 2001).

Asthma pathogenesis and progression are a multifactorial process in which social, environmental, and genetic factors interact. Health scientists and clinicians define asthma through observed adverse health effects corresponding to airway inflammation, obstruction, and remodeling [National Institutes of Health (NIH) 2003]. Occurring along a continuum, symptoms and reversibility vary among diagnosed individuals from mild to severe and are quantifiable by a range of biologic and clinical indicators (NIH 2003).

**Figure 2:**
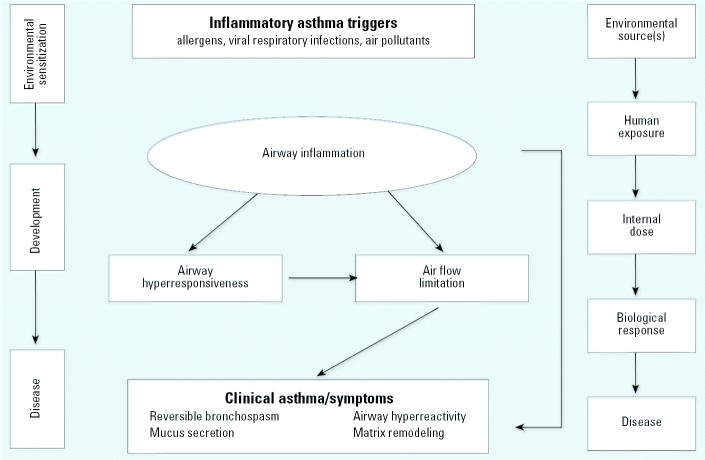
Asthma health effects and disease progression along the public health paradigm as they relate to environmental triggers and exposures. Adapted from Leikauf (2002), NIH (1997), and Sexton et al. (1995).

Asthma may be described as occurring in three stages—environmental sensitization, development, and disease—which appear in the left column of Figure 2 (Leikauf 2002). Each stage is associated with a set of biologic indicators, detailed in the middle section of Figure 2. Given its complexity, researchers identify asthma through multiple health end points, including clinical diagnosis, presence of high immunoglobulin (IgE) concentrations, and changes in lung capacity. For this reason, we place a high value on consistent use of clearly defined and quantifiable health end points for identifying asthmatics in our assessment. We consider those end points for the “disease” stage as the best available indicators meeting our evaluation criteria: reversible bronchospasms, airway hyperreactivity, mucus secretion, and matrix remodeling. According to the public health paradigm presented in Figure 2 (right), exposure to a toxicant may trigger a chain of biologic events that may ultimately lead to disease (Decaprio 1997; NRC 1994; Sexton et al. 1995).

Along all the stages of disease progression, scientists measure adverse effects associated with asthma through exposure/effect biomarkers, such as cytokine levels or changes in lung function. These are direct measurements indicating internal dose and biologic response. Additionally, susceptibility/genetic biomarkers measure and/or predict predisposition to these responses. Genetic biomarkers, such as up-regulation of genes, can provide quantifiable measures of exposure response in those predisposed to disease. Still, researchers continue to face great difficulty in identifying the most relevant genetic biomarkers for asthma. As a complex genetic disorder, asthma has multiple genetic loci, each contributing small to modest effects on overall disease progression (Xu et al. 2001). Hakonarson and Wjst (2001) reviewed > 150 linkage, association, and candidate gene studies collectively and report approximately 500 asthma and atopy loci identified across the genome, and additional gene identification continues to arise beyond these regions (Cookson 2003; Hakonarson et al. 2002; Holgate et al. 2003; Howard et al. 2003; Vercelli 2003; Weiss and Raby 2004). Finally, each gene may contain multiple single nucleotide polymorphisms associated with functionally different phenotypes.

To facilitate identification of key asthma candidate genes relevant to regulatory health science, we adapted the following criteria:

1. The gene product must be relevant to the pathophysiology of a clearly defined and consistent phenotype.
2. Gene function must be associated with exposure to a regulated pollutant or, at the very least, to a disease progression process known to be associated with exposure to the chosen regulated pollutant.
3. The mutation must be functionally relevant.
4. The magnitude of frequency of occurrence in the population must be measured and variation across populations (geography, race) must be considered.
5. There must be a high magnitude of association (i.e., preferably relative risk > 1.5) to an adverse health effect for the phenotype of interest.

Table 1 presents an illustrative set of candidate genes selected in light of the above criteria—interleukin (IL-4), IL-13, tumor necrosis factor-α (TNF-α), β_2_-adrenergic receptor (β_2_ADR), The β chain of the high affinity receptor for IgE, Fc ɛRI*-*β, and IL-4 receptor (IL-4R). The table’s columns represent the significant variables that asthma genetics research must address, in the form of the criteria outlined above. These columns contain the estimated relative risk of asthma associated with each polymorphism, based on studies comparing response between asthmatics and nonasthmatics and the hypothesized roles of the genes in asthma disease progression. The frequency in the population is also included. Not all candidate genes meet all of the above criteria.

It is important to note what is missing from Table 1. Because asthma research generating relative risks associated with specific polymorphisms and disease is still in its nascent stages, with many inconsistent results, none of these studies applies strictly to criterion 2 in that none relates directly to PM exposure. Still, Table 1 serves as an exemplary initial set of polymorphisms that generically apply to most air pollutants with known exacerbation of an associated adverse health effect. As research studies look more specifically at the effect of individual pollutants, a unique set of candidate genes and polymorphisms is expected for each pollutant.

We note that not all of the listed polymorphisms meet the relative risk baseline of 1.5 (criterion 5), because of the lack of refinement in association studies to date. Table 1 instead highlights relatively more robust studies using consistent phenotype and where polymorphisms show positive associations and fairly high frequencies in the population. The substantial variability in research associated with complete assessment of all candidate genes and their respective polymorphisms is not included. This variability is a result of inconsistencies in the analytical methods, varied definitions of phenotype, frequent lack of replication, and occasional lack of reported allelic frequencies and/or relative risks.

There are indications that one would find, in future studies of susceptible cohorts, increased relative risk of asthma based on gene–environment and gene–gene interactions. Two initial investigations suggest an increased relative risk from gene–gene interactions (Howard et al. 2002; Lee et al. 2004). These studies show that two cytokines, IL-4 and IL-13, interact with their shared receptor, IL-4R, to increase the relative risk for asthma from < 2 to as high as 3.54 and 4.87, respectively, in selected cohorts (Howard et al. 2002; Lee et al. 2004).

## Current Research Agendas Concerning PM, Asthma, and Genetics

As scientific understanding of the health effects from PM exposure improves, the U.S. EPA is required to ensure that the research necessary to protect asthmatics continues to progress. Section 103 of the 1990 CAAA mandates that the U.S. EPA establish, coordinate, conduct, and fund collaborative research (Figure 1, left column) (U.S. Clean Air Act Amendments 1990). Under an ideal application of this mandate, U.S. EPA administrators and research scientists would systematically identify and fund critical research, which would then serve as the basis for improving regulatory standards under the two-way feedback loop of the risk assessment/risk management paradigm.

PM exposure and health risk have been key regulatory foci in recent years (see Table 2). In 1997, Congress commissioned a National Academy of Science (NAS) committee on PM research and allocated nearly double the U.S. EPA’s requested PM research funding—totaling $49.6 million in fiscal year (FY) 1998 and $368 million between FY 1998 and FY 2003 (NRC 2004). The FY 1998 funding allowed the U.S. EPA to establish the five university-based PM centers and the National Environmental Respiratory Center.

Since 1998, the NAS committee has issued four substantial documents on PM research needs and developments. Among the top 10 research priorities cited, exposure of susceptible subpopulations and the increased risk for adverse health effects to these sub-populations are identified as warranting significant resource backing (NRC 1998, 1999, 2001, 2004). The current PM criteria document contains a toxicology subsection titled “Genetic Susceptibility to Inhaled Particles and Their Constituents” (U.S. EPA 2004a). The current PM staff paper states that “genetic susceptibility may play a role in differential responses to inhaled particles across a population” (U.S. EPA 2003). Still, the risk assessment process for PM NAAQS remains focused on epidemiologic research, particularly time-series and case–control exposure studies. Without additional research on asthma genetics, the opportunity to account for genetic susceptibility in the standard setting process will not be realized.

The research strategies and directions of an increasing group of government agencies present priorities and identify decision options en route to policy making that accounts for asthma genetics (Table 2). Many also include a multiyear funding allocation plan. For example, asthma genetics is high on the priority list of both the U.S. EPA’s Asthma Research Strategy and the DHHS/NIH’s Action against Asthma program (U.S. DHHS 2000; U.S. EPA 2002a). Working groups commissioned by governmental agencies, for example, the CDC, continue to stress their commitment to developing research on asthma genetics (Center for Genomics and Public Health 2004; Cunningham et al. 2003; Henry et al. 2002). Commitments such as this one promise the future development of air pollution exposure and risk assessments with regulatory relevance. Unfortunately, existing research strategies fail to specifically promote genetic susceptibility studies related to PM exposure at this time.

## Conclusions and Recommendations

This case study contributes an in-depth exploration of the potential role for genetic information in the regulatory framework under the CAA, specifically, in the process for developing the PM NAAQS. In this commentary, we develop the criteria by which one would select candidate genes relevant to regulatory health science, identify a specific statute (CAA) and key sections (103, 108, 112) where genetic information should be considered, and identify opportunities to improve decision making by incorporating information on genetic variability.

We propose criteria for selecting candidate genes to direct research on the etiology of asthma and its relevance to regulatory policy. Although we target PM and asthma, the discussion should be viewed more generally, because genetic information can substantially improve risk assessment on any environmental contaminant where genetic predisposition influences the risk of adverse health effects to identifiable subgroups. In a subsequent analysis (Cullen AC, Kramer CB, Faustman E, unpublished data), we extend this analysis to encompass an evaluation of genetic information in a decision analytic framework.

The present analysis further illustrates the multifactorial considerations necessary for devising adequate epidemiologic studies on asthma genetics. The establishment of guidelines for genetic research with regulatory relevance is imperative, given research trends in the study of complex disease. Recent analyses point out the imprecise replication of genetic association studies (Hirschhorn et al. 2002; Ioannidis et al. 2001; Merikangas and Risch 2003). Hirschhorn et al. (2002) reviewed 166 association studies and discovered a high level of consistent reproduction in only six polymorphisms across multiple diseases. Ioannidis et al. (2001) report similar results. These studies cite a range of complications that could be addressed by the development of appropriate guidelines. Despite these challenges, Merikangas and Risch (2003) support expansive research in molecular genetics, providing it is prioritized. Asthma genetics warrants a high-priority designation in the national research agenda given its association with factors beyond the exposed individual’s control and the strict health basis of criteria pollutant standard setting under the CAA.

As the U.S. EPA prepares to develop guidance on the inclusion of genomics information in risk assessment and decision making, we propose the following action items to encourage the pursuit of asthma genetics research solidly grounded in regulatory relevance:

- Fund research that specifically clarifies the role of genetic susceptibility factors in air pollution exposure. Although scientists continue to explore the role of genetics in asthma etiology, the U.S. EPA must ensure that epidemiologic and toxicologic studies are designed to provide a strong basis for this line of inquiry. Genetic information will become relevant to regulatory policy only with a solidly focused strategy.
- Fund longitudinal research with the aim of clarifying the complexity of air pollution effects over time and allowing for more complete evaluation of genetic biomarkers of susceptibility related to early adverse biologic effects.
- Develop strategies for the incorporation of genomic data in risk management methods. The *Interim Policy on Genomics* states, “EPA must understand how to develop and use the research tools made possible from genomics and understand the appropriate uses of genomics data to inform Agency decisions” (U.S. EPA 2002c). The policy cites the need to increase internal infrastructure, apply improved information technologies to analyze genomic data, and expand the capacity of computational toxicology into the future. Although all of these tools currently exist in a basic form, the U.S. EPA should augment them to meet their own needs as well as provide access to other individuals and organizations that will collaborate in this endeavor.
- Cooperate with other agencies on integration of research strategies. As mandated in Title I, section 103, of the CAAA (U.S. Clean Air Act Amendments 1990), the U.S. EPA must develop clear guidelines and objectives for interagency research and funding. Collaboration can ensure large population studies essential to genetic epidemiology, along with the development of widely accessible genetic databases. Without proper collaboration between U.S. EPA and other federal agencies, government-funded research projects may not produce population genetic science optimally relevant to regulatory policy, and/or future research may duplicate efforts already underway.
- Define validity of genetic biomarkers in measuring adverse health effects. Given the increased use and understanding of how biomarkers work as indicators for disease progression along the public health paradigm, the U.S. EPA should address the applicability of biomarkers for exposure and susceptibility (Decaprio 1997; Sexton and Adgate 1999). As the technology to quantify biomarkers becomes cheaper, easier, and more reliable, these indicators can be applied on a population scale (Decaprio 1997). The U.S. EPA should state the relevance of these biomarkers as indicators of adverse health effects to guide research and avoid litigation.
- Address ethical, legal, and social complexity. Many ethical and social considerations pertaining to genetic information currently lack legal interpretation or guidance. The U.S. EPA and partner agencies must assure the public that scientists will respect the special status of genetic information and use it ethically, so as not to invade privacy or improperly communicate risks. As suggested in the *Interim Policy on Genomics*, the U.S. EPA should be proactive in the implementation of guidelines for the use of genetic information (U.S. EPA 2002c, 2004b).

## Figures and Tables

**Table 1:** Examples of genetic and biologic factors contributing to asthma disease progression.

	Health effects	Genetic biomarkers	Hypothesized physiologic effect/phenotype	Polymorphism	Allelic frequency in population (OR or RR)	Estimated recessive impact on asthma (OR or RR)
Environmental sensitization	Allergic sensitization	IL-4	T_H_2 development (antigen mediated)	−590 C/T	0.17–0.80^a^,^b^	1.02–1.32^a^
		IL-13	Increased IgE secretion	−1111 T	0.21^c^	2.0^d^
Development	Inflammation and tissue damage	TNF-α	Enhanced inflammatory response	−308 G/A	0.22–0.30^e^	1.58–3.16^e^
		β_2_ADR	Damage smooth muscle	Gly 16	0.376^f^	1.77–3.03^f^
Disease	Asthma severity	IL-13	AHR, mucus production, fibrosis	−1111 T	0.21^c^	2.0^d^
	Bronchospasm	IL-4	T_H_2 cell development	−590 C/T	0.17–0.80^a^,^b^	1.02–1.32^a^
	Airway hyperreactivity	Fc ɛRI-β	IgE receptor, bronchial hyperresponsiveness	237 G	0.03–0.16^b^	2.3^b^
	Mucus secretion	β_2_ADR	Bronchoconstriction, airway hyperreactivity	Gly 16	0.376^f^	1.77–3.03^f^
	Matrix remodeling	IL-4R	Bronchial hyperresponsiveness	S 478 P	0.07–0.16^a^,^g^	0.86–1.13^a^

Abbreviations: OR, odds ratio; RR, relative risk; these are presented as single values or ranges, respectively. These candidate genetic biomarkers are characterized by their roles in allergic sensitization, inflammation, and tissue damage and/or disease symptomology. The genetic biomarkers were selected using criteria specific to regulatory health science. The IL-13 promoter polymorphism positions −1024 C/T, −1111 T, and −1055 T all have been shown to be identical using genetic analysis (Hummelshoj et al. 2003). We refer to this position as −1111 T-allele. Table adapted from Bracken et al. (2002) and Leikauf (2002).

a Lee et al. (2004).

b Zhu et al. (2000).

c Howard et al. (2001).

d Hummelshoj et al. (2003).

e Witte et al. (2002).

f Litonjua et al. (2004).

g Howard et al. (2002).

**Table 2:** Government agencies’ research strategies for addressing asthma genetic research.

Agency	Publication (date)	Example references to asthma genetics
U.S. EPA
	*Asthma Research Strategy* (U.S. EPA 2002a)	“[Asthma] has a definite genetic component” (p. 1) “Susceptibility Factors” ranked second in research priority after “Induction/Exacerbation” under “Prioritization of Research Areas” (p. 25) “Genetic Susceptibility” ranked second in research priority after “Exposure History” under “Susceptibility Factors” (p. 26)
	*Asthma toxics research strategy* (U.S. EPA 2002b)	“Genetic variation . . . define additional sensitive subpopulations” (p. 48)
	PM criteria document (October 2004; U.S. EPA 2004a)	Toxicology subsection: “Genetic Susceptibility to Inhaled Particles and Constituents” (sec. 7.5.2) Integrative Synthesis subsection, under Potentially Susceptible and Vulnerable Subpopulations, “Genetic Susceptibility (sec. 9.2.4.3)
	PM staff paper (draft, August 2003; U.S. EPA 2003)	“[A] number of new [toxicologic] studies . . . have suggested that genetic susceptibility may play a role in differential responses to inhaled particles across a population” (p. 3–67)
NAS
	*Research Priorities for Airborne Particulate Matter*, Vols. 1–4 (NRC 1998, 1999, 2001, 2004)	“[G]ene micro-array techniques are being used for studies of air pollutants even though determination of the most important genes, the roles of the genes and the best way to evaluate the huge amount of resulting data is still being resolved” (p. 114)
	*Clearing the Air: Asthma and Indoor Air Exposures* (Institute of Medicine 2000)	“As early as the 1920s, studies demonstrated that a familial pre-disposition to asthma existed, suggesting that genetics may play a role . . . however it explains only 30–80% of the asthma risk” (p. 28) “Furthermore, the interaction of different environment exposures with genetic susceptibilities must be elucidated” (p. 407)
DHHS
	*Action against Asthma* (U.S. DHHS 2000)	Research Priority Area One: determine the causes of asthma and develop interventions to prevent its onset “[A] major focus of research at several NIH institutes is on gene-environment interactions, and includes a genome-wide search as part of the Environmental Genome Project to identify genes that confer susceptibility to asthma” (p. 16) Urgent needs: primary prevention research (p. 16), study gene–environment interactions and links to characteristics of asthma
